# Whole‐Genome Sequencing Study in Koreans Identifies Novel Loci for Alzheimer Disease

**DOI:** 10.1002/alz.089543

**Published:** 2025-01-03

**Authors:** Moonil Kang, John J. Farrell, Congcong Zhu, Hyeonseul Park, Sarang Kang, Eun Hyun Seo, Kyu Yeong Choi, Gyungah R Jun, Sungho Won, Jungsoo Gim, Kun Ho Lee, Lindsay A. Farrer

**Affiliations:** ^1^ Department of Medicine (Biomedical Genetics), Boston University Chobanian & Avedisian School of Medicine, Boston, MA USA; ^2^ BK21 FOUR, Department of Integrative Biological Sciences, Chosun University, Gwangju Korea, Republic of (South); ^3^ Gwangju Alzheimer’s and Related Dementia (GARD) Cohort Research Center, Chosun University, Gwangju Korea, Republic of (South); ^4^ Premedical Science, College of Medicine, Chosun University, Gwangju Korea, Republic of (South); ^5^ Kolab Inc., Gwangju Korea, Republic of (South); ^6^ Department of Biostatistics, Boston University School of Public Health, Boston, MA USA; ^7^ Department of Ophthalmology, Boston University Chobanian & Avedisian School of Medicine, Boston, MA USA; ^8^ Alzheimer’s Disease Research Center, Boston University Chobanian & Avedisian School of Medicine, Boston, MA USA; ^9^ Institute of Health and Environment, Seoul National University, Seoul Korea, Republic of (South); ^10^ Department of Public Health Sciences, Graduate School of Public Health, Seoul National University, Seoul Korea, Republic of (South); ^11^ RexSoft Corps, Seoul Korea, Republic of (South); ^12^ Department of Biomedical Science, Chosun University, Gwangju Korea, Republic of (South); ^13^ Well‐ageing Medicare Institute, Chosun University, Gwangju Korea, Republic of (South); ^14^ Korea Brain Research Institute, Daegu Korea, Republic of (South); ^15^ Department of Neurology, Boston University Chobanian & Avedisian School of Medicine, Boston, MA USA; ^16^ Department of Epidemiology, Boston University School of Public Health, Boston, MA USA; ^17^ Boston University Chobanian & Avedisian School of Medicine, Boston, MA USA

## Abstract

**Background:**

With a rapidly aging population, South Korea anticipates a surge in Alzheimer disease (AD). However, the genetic basis of AD in Koreans is not well understood.

**Method:**

We sequenced the genomes of 3,540 Koreans (1,583 AD cases and 1,957 controls) older than age 60 and performed a genome‐wide association study (GWAS) of AD using logistic regression models that included covariates for age, sex, five ancestry principal components, and an empirical genetic relationship matrix. We also conducted association tests in subgroups stratified by *APOE ε*4 status or sex. Findings from the Korean sample were combined with results from two Japanese GWAS datasets using the inverse‐variance meta‐analysis method. We also performed gene‐based tests of aggregated rare variants in the Korean dataset. Novel findings were evaluated by pathway enrichment analysis and by differential gene expression analysis in brain tissue from controls and AD cases who were either cognitively impaired or healthy prior to death.

**Result:**

We identified genome‐wide significant (GWS) associations (*P* < 5.0 × 10^‐8^) with two well‐established AD loci (*APOE* and *SORL1*) in the total sample and with one novel locus, *ROCK2* (rs76484417, *P* = 2.71 × 10^‐8^) among *APOE ε*4 non‐carriers. Another novel study‐wide significant association (*P* < 2.47 × 10^‐6^) was found with aggregated rare variants in *MICALL1* (*P* = 9.04 × 10^‐7^). We also observed suggestive associations (*P* < 1.0 × 10^‐6^) with 14 novel loci (*MSX1*, *BCL11A*, *ARMH3*, *CTNNA3*, *YTHDF1P1*, *GRM3*, *LINC02479*, *LRCH1*, *COL9A1*, *TTC8*, *MCTP2*, *DLGAP2*, *TBC1D32*, and *EXD2*). Expression of several novel genes, including *ROCK2* and *MICALL1*, was significantly different in multiple cortical regions in AD cases compared to controls (*P* < 3.33 × 10^‐3^). *ROCK2* was also differentially expressed between AD cases with and without cognitive impairment (*P* = 1.34 × 10^‐4^).

**Conclusion:**

We identified GWS and suggestive association for AD with several loci in Koreans and Japanese not previously observed in other populations. Our results provide additional insight into genetic mechanisms leading to AD and cognitive resilience, as well as emphasize the need for further genetic studies of East Asian populations.

